# Compromised Bone Healing in Aged Rats Is Associated With Impaired M2 Macrophage Function

**DOI:** 10.3389/fimmu.2019.02443

**Published:** 2019-10-18

**Authors:** Julia Löffler, F. Andrea Sass, Sebastian Filter, Alexander Rose, Agnes Ellinghaus, Georg N. Duda, Anke Dienelt

**Affiliations:** ^1^Julius Wolff Institute and Center for Musculoskeletal Surgery, Charité-Universitätsmedizin Berlin, Corporate Member of Freie Universität Berlin, Berlin Institute of Health, Humboldt-Universität zu Berlin, Berlin, Germany; ^2^Berlin Institute of Health Center for Regenerative Therapies, Charité-Universitätsmedizin Berlin, Corporate Member of Freie Universität Berlin, Berlin Institute of Health, Humboldt-Universität zu Berlin, Berlin, Germany

**Keywords:** bone regeneration, macrophage, monocyte, CD14+ cells, aging, angiogenesis, compromised healing

## Abstract

Fracture repair is initiated by a multitude of immune cells and induction of an inflammatory cascade. Alterations in the early healing response due to an aged adaptive immune system leads to impaired bone repair, delayed healing or even formation of non-union. However, immuno-senescence is not limited to the adaptive immunity, but is also described for macrophages, main effector cells from the innate immune system. Beside regulation of pro- and anti-inflammatory signaling, macrophages contribute to angiogenesis and granulation tissue maturation. Thus, it seems likely that an altered macrophage function due to aging may affect bone repair at various stages and contribute to age related deficiencies in bone regeneration. To prove this hypothesis, we analyzed the expression of macrophage markers and angiogenic factors in the early bone hematoma derived from young and aged osteotomized Spraque Dawley rats. We detected an overall reduced expression of the monocyte/pan-macrophage markers CD14 and CD68 in aged rats. Furthermore, the analysis revealed an impaired expression of anti-inflammatory M2 macrophage markers in hematoma from aged animals that was connected to a diminished revascularization of the bone callus. To verify that the age related disturbed bone regeneration was due to a compromised macrophage function, CD14+ macrophage precursors were transplanted locally into the osteotomy gap of aged rats. Transplantation rescued bone regeneration partially after 6 weeks, demonstrated by a significantly induced deposition of new bone tissue, reduced fibrosis and significantly improved callus vascularization.

## Highlights

- Compromised bone regeneration in aged rats is connected to a reduced expression of the pan-macrophage markers CD14 and CD68.
- Anti-inflammatory M2 macrophage markers are decreased in the early callus from aged animals.
- Macrophage mediated angiogenesis is impaired in the early callus of aged animals.
- Transplantation of CD14 macrophage precursors rescues impaired bone regeneration in aged rats partially.

## Introduction

Fracture repair is a highly orchestrated process that involves a distinct pro- to anti-inflammatory signaling cascade in the hematoma, angiogenesis, coordinated extracellular matrix deposition and progression toward endochondral ossification (1). Comorbidities associated with an altered immune response, such as advanced age, diabetes, or rheumatoid arthritis, have been shown to reduce the initial biological potential of the fracture hematoma and may impair regeneration (2–4). In addition, disturbances in the revascularization or unbalanced expression of angiogenic growths factors can delay bone regeneration and eventually lead to the formation of atrophic pseudarthrosis (1, 5–8). Several studies report on the interconnection of immune cells, inflammation, and angiogenic processes. Especially monocytes and macrophages, cells from the innate immune system, are reported to regulate bone homeostasis and repair, as well as tissue vascularization (9, 10). In this context, optimal fracture repair is steered by a collaboration from infiltrating and bone-resident macrophages (11). In dependence of their surroundings, activated macrophages can adopt different functions that are characterized by a pro-inflammatory M1 (classically activated), and an anti-inflammatory M2 phenotype (alternatively activated). M1 macrophages produce large amounts of pro-inflammatory cytokines, as TNFa and IL-1b, and induce Th1 responses. In contrast, the so called M2 phenotype produces IL-10, IL-1 receptor type α, and TGF-β, induces Th2 immune responses, and contribute to angiogenesis, wound healing progression and granulation tissue maturation (12–16). Particularly M2 macrophages have shown to be highly diverse in their functionality and activation patterns. Over the last years, several M2 subsets have been identified (M2a, M2b, and M2c), even repolarization toward M1 phenotypes has been observed, highlighting the great plasticity of macrophages (17, 18).

However, detailed information on the diverse functions of even the more simplified concept of M1 and M2 macrophages during bone repair are rare. While Loi et al. demonstrated that a transition from M1 toward M2 phenotypes highly promotes bone formation *in vitro*, studies analyzing the impact of the different macrophage phenotypes in biologically compromised healing situations, as advanced age, are missing (19). We hypothesized that biologically impaired bone regeneration is connected to disturbances in macrophage functionality and alterations in the M1/M2 macrophage populations. To prove this hypothesis, we investigated the impact of M1/M2 macrophages on bone healing in aged rats more in detail.

## Materials and Methods

### Animal Studies

For the *in vivo* animal studies 3 and 12 month old female ex-breeder Sprague Dawley rats from Charles River WIGA Deutschland GmbH were used. These aged rats, that had a minimum of 3 L served as models for biologically impaired fracture healing that develop a non-union, when no additional treatment is applied (4, 20–22). Animal experiments were conducted in compliance with the ARRIVE guidelines and according to the policies and principles of the Animal Welfare Act, the National Institutes of Health Guide for the Care and Use of Laboratory Animals, and the National Animal Welfare Guidelines. All animal experiments were approved by the local legal representative (Institutional Animal Care and Use Committees, LaGeSo, G0120/14, G0172/15). Animals were anesthetized with 0.3 mg/kg Medetomidin DomitorH and 60 mg/kg Ketamin by intraperitoneal injection prior to surgical procedure. Additionally, 20 mg/kg Tramadol was administered as analgesia. Forty-five milligram per kilogram Clindamycin was administered by subcutaneous injection and eyes were prevented from drying out by application of eye balm. A longitudinal skin incision was made over the left femur. The bone was exposed by blunt fascia dissections. An in-house developed unilateral external fixator was mounted to stabilize the bone, made of stainless steel and titanium as published previously (20, 23). For an exact placement of the four wire holes, a drilling template was used for every procedure. After incision of the titanium wires, the external fixator bar was placed on the wires and a standardized 2 mm gap was sawn by osteotomy into the femoral bone. To ensure reproducibility of the gap size a sawing template was used at all times. Muscle fascia and skin were closed using absorbable and non-absorbable sutures, respectively. Animals received an anesthetic antagonist and were placed under red light until awakening. Post-surgical analgesia was given by addition of Tramadol (25 ml/l) to the drinking water for 3 days. Fracture healing was assessed after 3 and 7 days, as well as after 6 weeks by euthanization and femur dissection. Animal IDs, weights and group sizes can be found in Table 1.

**Table 1:** Animal numbers, weight, and group sizes for intraoperative cell transplantations.

**Animal ID**	**Weight (g)**	**Weight mean**	**Weight SD**	**Read-out**	**Group**	**Group size**
362	406	357	55.31	Histomorphometry/αSMA	PBMC	*n* = 5
364	326					
365	345					
369	288					
371	420					
381	407	375.3	66.14	Histomorphometry/αSMA	CD14+	*n* = 7
382	343					
385	384					
386	294					
387	318					
388	387					
390	494					
362	406	349.6	59.62	μCT	PBMC	*n* = 5
364	326					
366	308					
369	288					
371	420					
386	294	388.4	84.70	μCT	CD14+	*n* = 5
387	318					
388	387					
389	449					
390	494					

### Intraoperative Cell Transplantation

For the intraoperative cell transplantation of PBMCs or CD14+ cells into the osteotomy gap, 15 ml cardiac blood were drawn from 12-month-old donor rats. Subsequently, PBMCs were isolated by application of a density gradient using Histopaque-1083 (Sigma-Aldrich). The CD14+ subset was further extracted from the PBMC population using positive Magnetic Activated Cell Sorting by application of a murine CD14+ antibody (clone: biG, Abnova), combined with anti-mouse IgG microbeads from Miltenyi Biotec. Per blood clot 2 × 10^5^ of either PBMCs (including CD14+ cells) or CD14+ cells were re-suspended in 200 μl autologous blood, that was drawn just prior to the surgical procedure (including 10 μl sodium citrate, to prevent clotting). The osteotomy procedure was performed as described above. For cell transplantation groups, blood clotting was induced right before the clot was placed into the osteotomy gap by adding 7 μl CaCl_2_ 12%Thrombin (Baxter). The lid of a 1.5 ml Eppendorf tube served as a forming device for the artificial hematoma. The clot was designed to exactly fit into the osteotomy gap (same height as gap width), but with a slightly larger diameter, to ensure that the osteotomy gap was spanned by the clot. No differences in clot quality/nature were observed at any point. It was previously shown, that bone formation after a regular healing time of 6 weeks in animals receiving an empty autologous blood clot without additional cell supplementation is comparable to the one seen in animals that received a PBMC supplemented blood clot (24, 25) thus PBMC supplemented artificial hematoma was used as control in this study rather than the autologous blood clot alone.

### μCT

Bone healing was assessed *in vitro* with micro-computed tomography on formaldehyde-fixed left femurs extracted from euthanized animals 6 weeks after surgery. A region of interest (ROI) covering the 2 mm osteotomy gap plus 1 mm proximal and distal was scanned in a Viva CT 40 microCT (Scanco Medical AG) with application of a voxel size of 10.5 μm, 55 keVp, 145 μA. Bone microstructure trabecular number, trabecular space, trabecular thickness) and the key parameters of tissue and bone mineral content were assessed using the respective software from the device supplier (Scanco Software, Scanco Medical AG). 3D μCT reconstruction were done using CTvox (version 3.2.0.r1294). All analyses were performed in a blinded manner by two different observers with automatically assignment of μCT screen-numbers, to avoid bias to the treatment.

### Fracture Hematoma Extraction and Gene Expression Analysis

For gene expression analysis, femurs were excised from animals euthanized 3 and 7 days after osteotomy. Surrounding muscle tissue was dissected and tissue containing the fracture callus plus 1 mm proximal and distal to the osteotomy gap was extracted and immediately transferred to liquid nitrogen. Subsequently, tissue samples were pestled while frozen in liquid nitrogen and collected in TRIzol Reagent (LifeTechnologies) afterwards. RNA was isolated according to the manufacturer's protocol, followed by determination of RNA concentration using a Nano-Drop spectrophotometer. cDNA was transcribed from 25 ng/μl RNA with iScript reverse transcriptase as indicated by the manufacturer (Bio-Rad Laboratories GmbH) and gene expression was determined via quantitative real time PCR (iQ5 Cycler, Bio-Rad Laboratories GmbH). Primer sequences (Table 2) were generated using the primer 3 web based software (http://primer3.ut.ee/) and tested for specificity (ePCR, http://www.ncbi.nlm.nih.gov/tools/epcr/). Expression of each gene was calculated according to the ddCT method with adjustment for primer efficiency and normalization to TATA-box binding protein (Tbp) expression by utilizing the REST software (26). The housekeeping gene Tbp was tested against others (Gapdh, Actb, Eif4e, B2m) and was found to be the most stable gene in all investigated samples.

**Table 2:** Primer sequences.

**Gene**	**Gene name**	**Forward 5^**′**^-3^**′**^**	**Reverse 5^**′**^-3^**′**^**
CD14	Cluster of differentiation 14	aactgaagcctttctcggagc	gcataagcttcatggtcggt
CD68	Cluster of differentiation 68	tccagcaattcacctggacc	aagagaagcatggcccgaag
CD80	Cluster of differentiation 80	gctgctggttggtcttttcc	ttcttgtactcgggccacac
CCR7	C-C chemokine receptor type 7	tacatcggcgagaacaccac	caggacttggcttcgctgta
CD163	Cluster of differentiation 163	ctggagcatgaacgaggtgt	ttcctgagcatcggttgtcc
CD206	Cluster of differentiation 206	cagtttgagggcagcaagag	acactcaggagctcagcatt
Tie-2	TEK tyrosine kinase; angiopoetin receptor	tctgctctcaaggatggcaa	cacactgcagacccaaactc
Dectin	C-type lectin domain family 7 member A (CLEC7A)/Dectin	cgtcttttctggaccttgcc	acggcccttcactctgattg
PDGFα	Platelet derived growth factor alpha	ttgaacatgacccgagcaca	acacctctgtacgcgtcttg
PDGFRα	Platelet derived growth factor receptor alpha	agtgcttggtcggatcttgg	gagcatcttcacagccacct
PDGFβ	Platelet derived growth factor beta	ttgaacatgacccgagcaca	acacctctgtacgcgtcttg
PDGFRβ	Platelet derived growth factor receptor beta	cgttgcaggtggtgtttgag	acacggacagggacattgac
HIF-1α	Hypoxia-inducible factor 1, alpha subunit	tcatcagttgccacttcccc	actgggccatttctgtgtgt
VEGF	Vascular endothelial growth factor	aaagcccatgaagtggtgaa	tctgcatagtgacgttgctc
VEFGR	Vascular endothelial growth factor receptor	agaacagagctcaacgtggg	atctttgccacagtcccagg

### Histological Analysis

Histological analyses were performed on frozen section according to the Kawamoto's film method (27). Excised femurs were fixed in a 4% PBS/PFA solution for 24 h at 4°C. For cryo-protection purposes, femurs were transferred into 10, 20, 30% sucrose solutions in ascending order for 24 h at 4°C at a time, subsequently embedded in SCEM-Medium and frozen by immersion into cold n-Hexan. Embedded and frozen samples were sectioned into 5 μm thick slices. All femurs were oriented in the same manner for the histological analyses, where the proximal part of the femur is placed on the left and the distal end of the femur on the right side of the image.

To distinguish between different calcified and soft tissues Movant Pentachrome staining was used. Prior to the staining procedure, slides were fixed with 4% PFA/PBS for 15 min. Five subsequent stainings are applied to stain mineralized bone (yellow/orange), collagen (yellow), cartilage (green/blue), osteoid (dark red), elastic fibers (orange/red), and nuclei (blue-black). After fixation samples were rehydrated for 5 min before alcian blue staining was applied for 30 min, which targets acid proteoglycans structures like chondroitin sulfate. One hour incubation in alkaline ethanol, stabilizing the blue-green pigment. Afterwards, slides were incubated in Weigert's Iron hematoxylin solution (15 min), to stain nuclei. Cell plasma was stained by brilliant crocein acid fuchsine (15 min), followed by differentiation in 0.5% acetic acid. As a last step, slides were incubated in phosphotungstic acid (20 min) and the connective tissue was stained by saffron du gatinais solution.

Vascularization analysis was performed on smooth muscle actin (αSMA) immunohistochemical staining. Slides were fixed with 4% PFA/PBS prior to the staining procedure. All subsequent staining steps were carried out in a humid chamber, at room temperature, unless stated otherwise. To block against unspecific background, samples were incubated with 2% normal horse serum before overnight application of the primary antibody (α-SMA, Dako M0851) at 4°C. Thirty minutes incubation with the secondary antibody (biotinylated anti-mouse IGG, rat-adsorbed, made in horse) and subsequent application of AB complex (ABC-AP Vectastain Kit—SP 5000) for 50 min. 2 × 5 min incubation with chromogen buffer before visualization of the vessels with AP- substrate (Red AP Substrate Kit, Vector—SK 5100). Color development was controlled under the microscope and ended by washing the slides with PBS. Nuclei were counter stained using Mayers Hämalaun for 1.5 min, unstained surrounding tissue is blued by tab water.

Pictures of the stained samples were taken with Zeiss Axioscope 40 Microscope, 10 × objective (plus condenser) and the corresponding Imaging AxioVision LE Software (Carl Zeiss). Tissue quantification was done with ImageJ (Version 1.44p; http://rsbweb.nih.gov/ij/) using a semi-automated method on blinded sections. Vessels were counted manually in a blinded approach. Inclusion criteria included a clear endothelial cell border, a visible lumen and non-muscle association. A region of interest (ROI) including the osteotomy gap and 1 mm proximal and distal to it was investigated.

### Statistics

Determined values are depicted as bar charts showing mean ± standard deviation. For statistical analysis SigmaPlot 11.0 was used. Data were checked for normality distribution and analyzed with Student's *t*-test or ANOVA using a Bonferroni correction. If normality distribution could not be confirmed, data were analyzed using a non-parametric Man Whitney U Test or a multiple pairwise comparison according to Dunn's method. A *p* ≤ 0.05 was considered as significant. Each analysis was performed with three technical replicates per biological samples. The applied statistical method and the amount of individual biological samples (n) that were analyzed are indicated in the respective figure legends.

## Results

### Diminished Macrophage Accumulation in Bone Callus of Aged Rats

To validate our hypothesis, we analyzed the expression of commonly applied macrophage markers at early fracture healing time-points (28–32). Therefore, we extracted hematoma/callus tissue 3 and 7 days after osteotomy from young (3 months) and aged (12 months), female rats. The aged animals served hereby as a model system for impaired bone regeneration that develop a non-union without additional treatment, as we have shown previously (4, 20–22).

**Figure 1:**
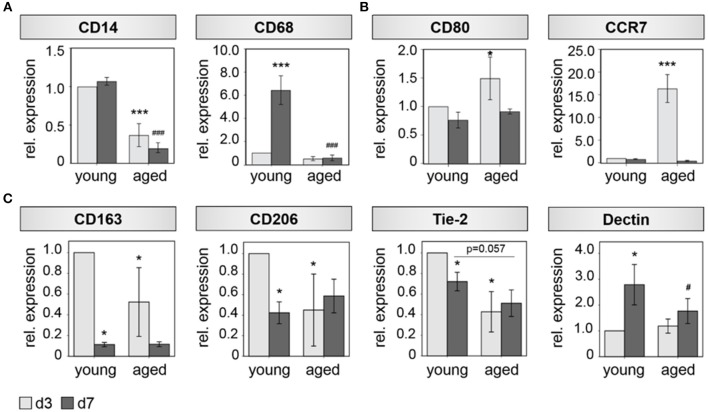
M2 Macrophage function is impaired in bone hematoma of aged rats. **(A)** The general monocyte maker CD14 and the general mocrophage maker CD68 show a significant reduced expression 3 and 7 days after osteotomy in hematoma tissue extracted from aged animals compared to young ones. **(B)** The makers CD80 and CCR7 that are predominantly expressed by M1 macrophages show higher expression levels in fracture callus tissue from aged animals at day 3. **(C)** Marker genes that are mostly expressed by M2 macrophages (CD163, CD206, Tie-2, Dectin) show a significantly diminished expression in hematoma tissue from aged animals compared to the expression found in fracture hematoma extracted from young animals. *n* = 4–5, ^*^significant to young d3, ^*^p < 0.05; ^**^p < 0.001; ^#^significant to young d7, ^#^*p* < 0.05; ^###^*p* < 0.001, ANOVA.

Interestingly, a diminished expression of monocyte/ macrophage related genes was detected in bone callus tissue derived from aged animals, which develop a non-union when no additional treatment is applied. The expression of the monocyte/macrophage precursor marker CD14 and the general macrophage marker CD68 were significantly downregulated in bone callus tissue of aged animals, when compared to their expression in callus tissue derived from young animals (Figure 1A). Next, we investigated the expression of specific M1 and M2 macrophage polarization markers in more detail, considering the various processes, in which macrophage subsets take part during healing cascades (10, 33). The M1 markers CD80 and CCR7 showed a significant upregulation in fracture callus tissue of aged animals compared to young animals, at day 3 after osteotomy (Figure 1B). We further detected significant alterations, when we investigated the expression of M2 specific markers (Figure 1C). CD163, CD206, and Tie-2 showed lower expression levels in bone callus tissue derived from aged animals compared to younger ones (Figure 1C). These differences reached significance on day 3 after osteotomy. The M2 marker Dectin on the other hand, showed an upregulation from day 3 toward day 7 after osteotomy in young and aged animals. However, its expression was significantly lower in callus tissue from aged compared to young animals (Figure 1C). When investigating unfractured contralateral bone tissue no significant changes in marker gene expression levels could be detected (Supplementary Figures 1A,B and Supplementary Table 1).

### Disturbed Callus Revascularization in Aged Rats

**Figure 2:**
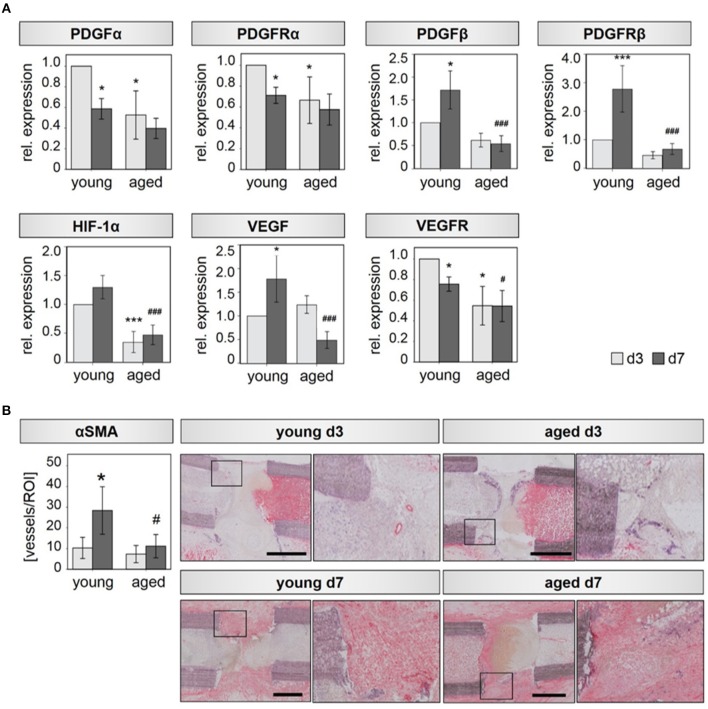
Bone callus vascularization is compromised in aged rats. **(A)** Expression of the angiogenic transcription factor HIF-1α, the angiogenic growth factors PDGFα and VEGF and their respective receptors PDGFRα, PDGFRβ, and VEGFR is significantly reduced in callus tissue from aged rats extracted 3 and 7 days after osteotomy. *n* = 4–5, ^*^significant to young d3; ^#^significant to young d7, ANOVA on Ranks. **(B)** Vessel number evaluated in αSMA stained tissue sections. For each condition, the left picture shows the overview of the whole region of interest, the right picture shows the magnification of the region within the square–found in the respective overview mage. Femurs are placed in the same orientation, with the proximal side on the left and distal on the right side. y/d3, y/d7 *n* = 5, a/d3 *n* = 3, a/d7 *n* = 6, ^*^significant to young d3, ^*^p < 0.05, ^***^p < 0.001; ^#^significant to young d7, ^#^*p* < 0.05, ^###^*p* < 0.001, ANOVA.

M2 macrophages are known to be highly involved in the regulation of angiogenic responses (34, 35). We therefore hypothesized, that the altered M2 macrophage expression profile detected in bone callus of aged animals is further accompanied with impaired revascularization of the injured bone tissue. To this end, we investigated the expression of several pro-angiogenic (growth) factors and their corresponding receptors. Indeed, we found a significantly downregulated expression of PDGFα, PDGFRα, HIF1α, and VEGFRα in fracture callus tissue of aged animals 3 days after osteotomy compared to tissue harvested from young ones (Figure 2A). PDGFβ and its receptor PDGFRβ did not reach statistical significance but showed the same trend of lower expression levels in fracture calli from aged animals compared to young animals at day 3 (Figure 2A). Significantly lower expression of HIF1α and VEGFRα was still evident 7 days after osteotomy in aged rats. In addition, expression levels of PDGFβ, PDGFRβ, and VEGF were significantly reduced at day 7 when comparing aged animals to young ones (Figure 2A). The analyzed genes showed no significant regulation in expression levels when investigating control tissue (Supplementary Figure 1C).

The decreased expression of angiogenic growth factors in aged animals is also reflected by a diminished number of newly forming vessels, identified by immunohistochemical assessment. Aged rats displayed 1.4-fold reduced numbers of alpha smooth vessel actin (αSMA) positive vessels in the callus region compared to young animals at day 3. At day 7 αSMA positive vessel numbers were reduced by a factor of 2.5 in aged animals (Figure 2B). Vessel diameter was also significantly reduced in hematoma tissue derived from aged animals at day 7 (Supplementary Figure 2).

### Monocyte Transplantation Rescues Impaired Bone Healing in Aged Rats

Based on the findings discussed above, we assumed that a diminished monocyte number and possibly decreased M2 macrophage differentiation or the lack of a shift from M1 to M2 population may lead to the observed delayed healing. Enrichment of the naturally occurring monocyte/macrophage CD14+ precursor cells in the osteotomy gap region of aged animals may assist in steering a successful endogenous healing cascade. Thus, local transplantation of CD14+ cells, could improve the impaired bone regeneration observed in aged animals.

**Figure 3:**
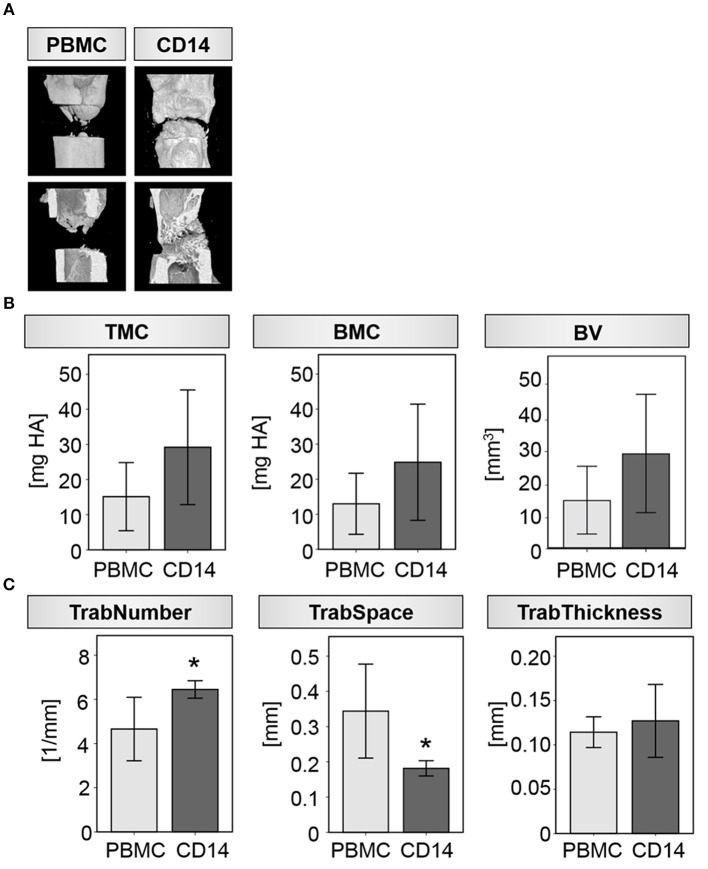
Transplantation of CD14+ macrophage precursors partly rescues impaired bone regeneration in aged rats. **(A)** Exemplary 3D reconstructions from four individual animals receiving either PBMC or CD14+ cells locally at the osteotomy site. Bone healing was induced after CD14+ cell transplantation. **(B)** Mineral deposition was prone to be increased after CD14+ cell transplantation in the investigated ROI. **(C)** Formation of new trabeculae was significantly induced after CD14+ cell transplantation as indicated by an increased number and a reduced space between them. Thickness of the single trabeculae were unaffected by cell transplantation. *n* = 5, ^*^significant to PBMC, *p* < 0.05, *t*-test.

Indeed, upon local transplantation of an artificial blood clot containing CD14+ cells into the fracture gap of aged animals directly after osteotomy-induced trauma, induction of new bone formation was detected when compared to the control group (artificial blood clot containing PBMCS) (Figure 3). Reconstructions from μCT analysis showed a clear formation of new bone tissue within the osteotomy gap (Figure 3A), connected to a quantitatively increased mineral deposition in the CD14+ enriched transplantation group compared to the PBMC control group (Tissue Mineral Content-TMC, Bone Mineral Content-BMC and Bone Volume-BV) (Table 3; Figure 3B). When we investigated the microstructure of the newly formed bone more in detail, we detected a significant increase in trabecular number and a significantly diminished space between the trabeculae (Table 3; Figures 3A,C).

**Table 3:** μCT investigations 6 weeks after cell transplantation.

	**PBMC**	**CD14**
TMC [mg HA]	15.13 ± 9.70	29.19 ± 16.32
BMC [mg HA]	12.99 ± 8.71	24.84 ± 16.58
BV [mm^3^]	14.95 ± 10.60	29.51 ± 18.50
Trabecular number [1/mm]	4.66 ± 1.44	6.45 ± 0.40
Trabecular space [mm]	0.34 ± 0.13	0.18 ± 0.02
Trabecular thickness [mm]	0.11 ± 0.02	0.13 ± 0.04

**Figure 4:**
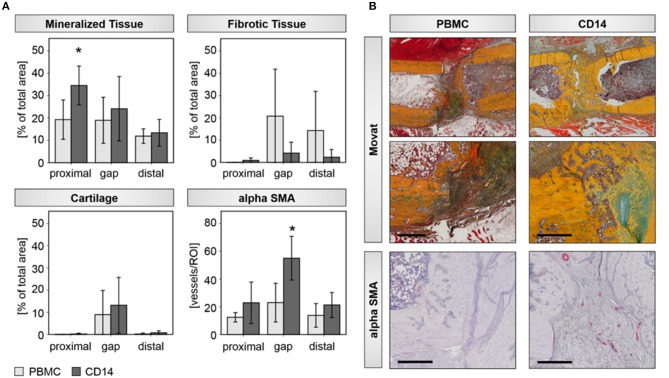
Histological assessments verify improved bone healing after CD14+ cell transplantation aged rats. **(A)** Histomorphometric evaluations of mineralized, fibrotic, and cartilage tissue with the osteotomy gap, as well as distal and proximal to it, were done. The analysis revealed a significant induced deposition of mineralized bone matrix and by trend a diminished formation of fibrotic tissue, when aged animals received CD14+ cells locally at the osteotomy site. Number of αSMA positive vessel increased significantly in the region of interest after CD14+ cell transplantation. PBMCs *n* = 5, CD14+ *n* = 7, ^*^significant to PBMC, *p* < 0.05. **(B)** Exemplary pictures of Movat-Pentachrom (mineralized bone, yellow/orange; collagen, yellow; cartilage, green/blue; osteoid, dark red; elastic fibers, orange/ref; nuclei, blue-black) and aSMA stained tissue sections. Femurs are placed in the same orientation, with the proximal side on the left and distal on the right side. Bar = 500 μm.

Histological evaluations confirmed the findings from the radiological analysis. A significant induction of new bone tissue mainly proximal to the bone trauma and partly within the gap region was detected in the CD14+ transplantation group (Table 4; Figures 4A,B). By trend, we also found a decreased formation of fibrous tissue, when CD14+ cells were transplanted in aged animals (Table 4; Figures 4A,B). Interestingly, the improved bone tissue regeneration after CD14+ cell transplantation was accompanied with a significant induction of new vessel formation (Table 4; Figures 4A,B).

**Table 4:** Histomorphometric evaluations 6 weeks after cell transplantation.

	**PBMC**			**CD14**		
	**Proximal**	**Gap**	**Distal**	**Proximal**	**Gap**	**Distal**
Mineralized tissue [% of total area]	19.27 ± 8.81	18.96 ± 10.28	11.89 ± 3.26	34.54 ± 8.65	24.13 ± 14.38	13.39 ± 5.96
Fibrotic tissue [% of total area]	n.d	20.79 ± 21.10	14.29 ± 17.60	0.91 ± 1.12	4.18 ± 4.88	2.34 ± 3.46
Cartilage [% of total area]	0.01 ± 0.03	8.95 ± 10.88	0.21 ± 0.47	0.20 ± 0.38	13.18 ± 12.55	0.75 ± 0.92
Vessel number [1/ROI]	12 ± 3	23 ± 14	14 ± 9	23 ± 15	55 ± 16	21 ± 9

## Discussion and Conclusion

Macrophages are essential for bone regeneration and they participate in all stages of healing, promote collagen I deposition and matrix mineralization by osteoblasts *in vitro* and *in vivo* (9, 36, 37). Additional evidence that macrophages play important roles during bone regeneration can be obtained from studies reporting on systemic depletions of macrophages in experimental mouse models. Independent of the time point of macrophage depletion, a diminished formation of new bone matrix and an altered endochondral ossification process is visible 7–28 days after injury (36, 38).

The M1 to M2 switch plays a vital role during healing progression as reported recently by co-workers from our institute and others (19, 38). We confirm here that macrophage activity plays a significant role in bone repair and that an imbalance in the M1/M2 macrophage differentiation is associated with disturbed bone regeneration in aged rats. Furthermore, we showed that local transplantation of macrophage precursors can enhance and potentially rescue bone repair under biologically impaired conditions, presumably by an induction of M2 macrophage differentiation. Our findings highlight the potential of local cell transplantations, here monocytes/macrophages to steer bone repair, especially under compromised conditions, since we used macrophage precursors derived from circulating blood of aged matched donors for local cell transplantation, which presumably shifted the endogenous cell balance and thus promoted healing. Ongoing research further highlights a close connection between macrophages and osteogenic differentiation. A recent study, reported on the osteogenic differentiation capacity of MC3T3 pre-osteoblasts in *in vitro* co-cultures with macrophages (19). When MC3T3 cells were cultivated together with M1 macrophages that underwent an IL-4 triggered M2 switch during MC3T3 osteoblast maturation, mineralized matrix deposition was significantly induced (19). This might be due to M2 macrophage-induced BMP-2 secretion, which is a major contributor to osteogenic differentiation (39). In addition, recent studies from Gibon et al. support our hypothesis of a disturbed M1/M2 phenotype balance in aged. They could show that bone marrow macrophages isolated from aged mice have a higher pre-activated resting state and increased expression of the pro-inflammatory cytokine TNFa after activation than macrophages isolated from young animals (40). Furthermore, they described an impaired M2 polarization of bone marrow macrophages derived from aged mice (40). Recently Vi et al. also reported that the age of macrophages is crucial for fracture repair. Parabiosis and fractionated bone marrow transplantation experiments in mice showed that young macrophages can rejunvenate healing potential of old bone marrow stromal cells, while old macrophages impair healing of young bone marrow stromal cells (41).

There is also *in vivo* evidence that M2 rather than M1 macrophages regulate bone healing. Animals that were administered with CSF-1, which is required for macrophage differentiation, have an increased abundance of M2 macrophages within the fracture site and show improved healing outcomes (36, 42). In addition, M2 macrophages are highly pro-angiogenic and secrete various growth factors, as e.g., TGF-b, TGF-a, bFGF, PDGF, and VEGF (43, 44). Thereby they might regulate revascularization and matrix maturation of the callus tissue. The angiogenic capacity of monocytes and their descendent macrophages is also proven by investigations of Ccr2^−/−^ mice. Beside compromised cartilage maturation, Ccr2^−/−^ mice show an impaired formation of new blood vessels within the fracture site (45). Moldovan et al. reported that the angiogenic capacity of macrophages relates to their capability to degrade extracellular matrix. Using a transgenic mouse model of ischemic cardiomyopathy, where monocytes were attracted to the myocardium by the targeted overexpression of CCL2, they showed tunnel carving by macrophages, which provide growing vessels with a path for invading capillaries (12).

However, there is still an ongoing discussion on the state of macrophage polarization and activation and its effect on bone regeneration. M1 macrophages for instance can be beneficial or deleterious for bone formation, highly depending on the study design as recently reviewed by Pajarinen et al. (9). Another possibility is that all macrophage phenotypes can promote osteogenesis, but that their effectiveness is connected to different physiological and pathophysiological states (9).

Related to these recent reports and the current study, it is still a matter of discussion whether the pro-regenerative function of M2 macrophages in bone repair is related to their angiogenic properties and/or their ability for matrix degradation and how these characteristics may be effected by compromised biological conditions. While our work gives new insights concerning the beneficial effect of local macrophage enrichment on bone healing outcome, it is limited in showing M1/M2 dynamics early after transplantation. Additional research is needed to explore the exact role of M2 macrophages and other macrophage phenotypes in the early healing cascade.

## Data Availability Statement

The datasets generated for this study are available on request to the corresponding author.

## Ethics Statement

The animal study was reviewed and approved by Institutional Animal Care and Use Committees, LaGeSo, G0120/14, G0172/15.

## Author Contributions

AD has participated in conception and design of the study, acquisition, analysis and interpretation of data, and manuscript writing and editing. JL has contributed to data acquisition, analysis and interpretation of data, and manuscript writing and editing. AE, AR, FS, and SF have participated in acquisition, analysis and interpretation of data, and manuscript editing. GD contributed to conception and design of the study, interpretation of data, and manuscript editing. All co-authors approved the final version of the submitted manuscript.

### Conflict of Interest

The authors declare that the research was conducted in the absence of any commercial or financial relationships that could be construed as a potential conflict of interest.
