# Supplementary material for: Compromised Bone Healing in Aged Rats Is Associated With Impaired M2 Macrophage Function
Source: Front Immunol. 2019 Oct 18;10:2443. doi: 10.3389/fimmu.2019.02443 (PMC6813416; doi:10.3389/fimmu.2019.02443)
Supplement: Supplementary file 1 [file Data_Sheet_1.docx]

Supplementary Material

#
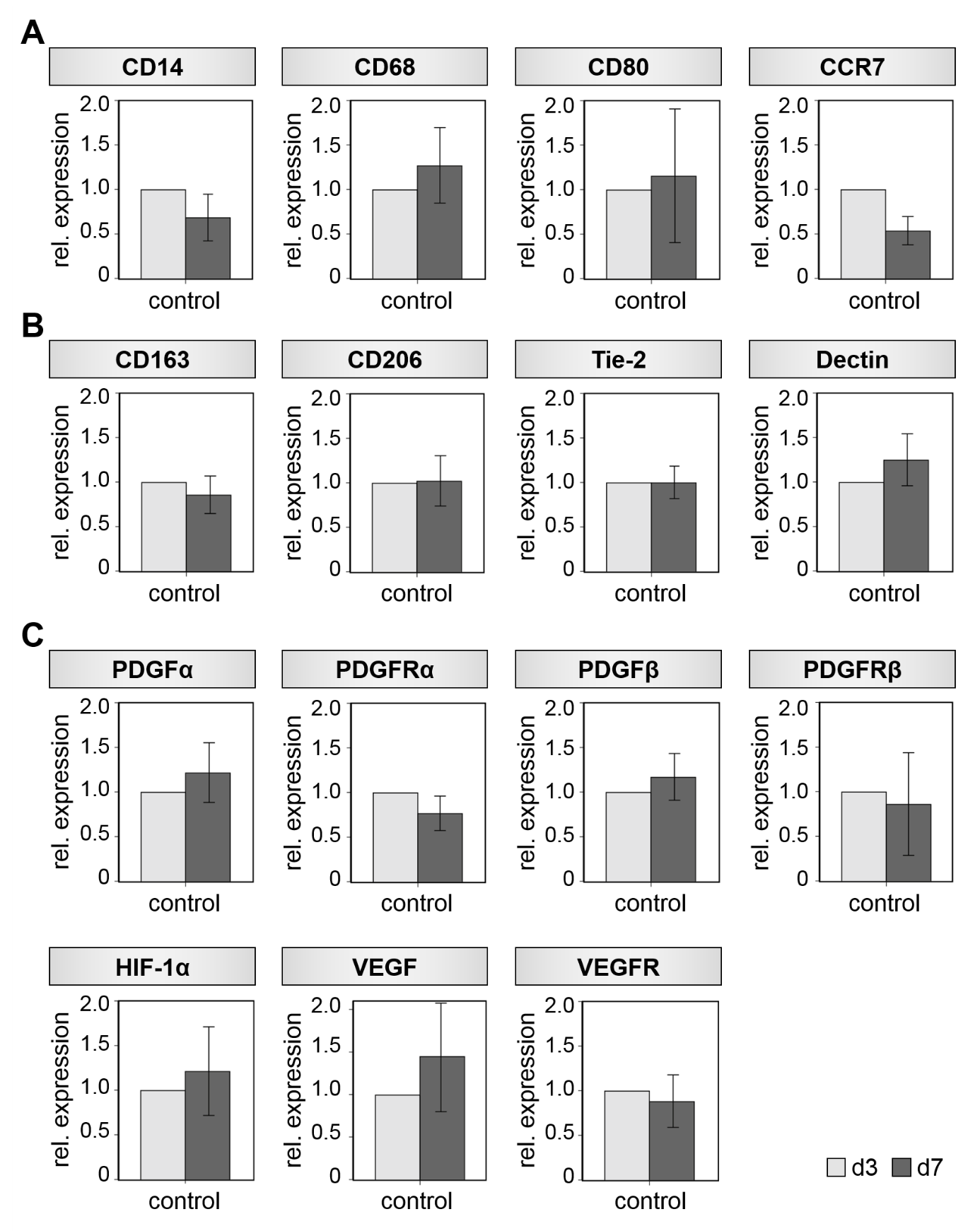
Supplementary Figures

**(A)**

**(B)**

**(C)**

# Supplementary figure 1. Gene expression of unfractured control bone. (A) Monocyte/macrophage and M1 macrophage gene expression show no significant changes between day 3 and day 7. (B) Marker genes for M2 macrophages and angiogenic macrophages show no difference between d3 and d7 expression. (C) No significant difference in expression was detected for angiogenic genes between d3 and d7 unfractured control tissue. Control tissue from aged and young animals was pooled and analyzed. The unfractured contralateral femoral bone served as control tissue. n=2 for CCR7, n=4-5 all other genes, ANOVA on ranks.


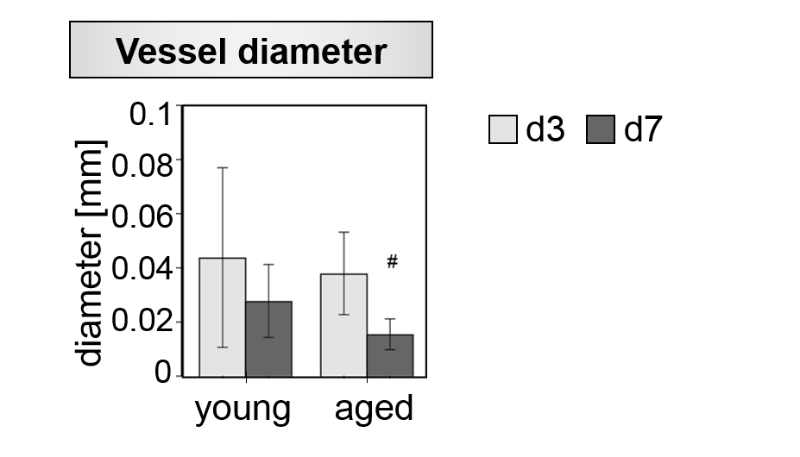


**Supplementary figure 2. Vessel diameter of identified vessels within the fracture gap region of interest**. Day 3 conditions showed little number of vessels, which showed no difference in lumen diameter between young and aged animals. At day 7 vessels identified in young animals showed a greater diameter, when compared to aged individuals. Mean ± SD, y/d3, y/d7 n=5, a/d3 n=3, a/d7 n=4, # significant to young d7, #-p-value <0.05, ANOVA.

***Supplementary table 1: In vitro investigations of macrophage and angiogenic markers in fracture hematoma extracted 3 and 7 days after osteotomy.***

| **Gene/condition** | **Day 3** | **Day 7** |
| --- | --- | --- |
| CD14 | 1.00±0.00 | 0.69±0.26 |
| CD68 | 1.00±0.00 | 1.27±0.42 |
| CD80 | 1.00±0.00 | 1.16±0.75 |
| CCR7 | 1.00±0.00 | 0.54±0.16 |
| CD163 | 1.00±0.00 | 0.86±0.21 |
| CD206 | 1.00±0.00 | 1.02±0.28 |
| Tie-2 | 1.00±0.00 | 1.00±0.18 |
| Dectin | 1.00±0.00 | 1.25±0.29 |
| PDGFα | 1.00±0.00 | 1.22±0.33 |
| PDGFRα | 1.00±0.00 | 0.77±0.19 |
| PDGFβ | 1.00±0.00 | 1.17±0.26 |
| PDGFRβ | 1.00±0.00 | 0.86±0.57 |
| HIF-1α | 1.00±0.00 | 1.21±0.50 |
| VEGF | 1.00±0.00 | 1.45±0.64 |
| VEGFR | 1.00±0.00 | 0.88±0.29 |
